# Iron oxide nanozymes stabilize stannous fluoride for targeted biofilm killing and synergistic oral disease prevention

**DOI:** 10.21203/rs.3.rs-2723097/v1

**Published:** 2023-04-03

**Authors:** Yue Huang, Yuan Liu, Nil Pandey, Shrey Shah, Aurea Simon-Soro, Jessica Hsu, Zhi Ren, Zhenting Xiang, Dongyeop Kim, Tatsuro Ito, Min Jun Oh, Christine Buckley, Faizan Alawi, Yong Li, Paul Smeets, Sarah Boyer, Xingchen Zhao, Derk Joester, Domenick Zero, David Cormode, Hyun Koo

**Affiliations:** University of Pennsylvania; Biofilm Research Labs, Levy Center for Oral Health, School of Dental Medicine, University of Pennsylvania, Philadelphia, PA, USA; University of Pennsylvania; University of Pennsylvania; University of Pennsylvania; University of Pennsylvania; University of Pennsylvania; University of Pennsylvania; Jeonbuk National University; Biofilm Research Labs, Levy Center for Oral Health, School of Dental Medicine, University of Pennsylvania, Philadelphia, PA, USA; University of Pennsylvania; Indiana University; Department of Cariology, Operative Dentistry and Dental Public Health, Oral Health Research Institute, Indiana University School of Dentistry, Indianapolis, USA; Biofilm Research Labs, Levy Center for Oral Health, School of Dental Medicine, University of Pennsylvania, Philadelphia, PA, USA; Northwestern University; Northwestern University; Northwestern University; Northwestern University; Department of Cariology, Operative Dentistry and Dental Public Health, Oral Health Research Institute, Indiana University School of Dentistry, Indianapolis, USA; University of Pennsylvania; University of Pennsylvania

**Keywords:** fluoride, nanoparticles, biofilm, dental caries, enamel ultrastructure, microbiome, ROS

## Abstract

Dental caries (tooth decay) is the most prevalent human disease caused by oral biofilms, affecting nearly half of the global population despite increased use of fluoride, the mainstay anticaries (tooth-enamel protective) agent. Recently, an FDA-approved iron oxide nanozyme formulation (ferumoxytol, Fer) has been shown to disrupt caries-causing biofilms with high specificity via catalytic activation of hydrogen peroxide, but it is incapable of interfering with enamel acid demineralization. Here, we find notable synergy when Fer is combined with stannous fluoride (SnF_2_), markedly inhibiting both biofilm accumulation and enamel damage more effectively than either alone. Unexpectedly, our data show that SnF_2_ enhances the catalytic activity of Fer, significantly increasing reactive oxygen species (ROS) generation and antibiofilm activity. We discover that the stability of SnF_2_ (unstable in water) is markedly enhanced when mixed with Fer in aqueous solutions without any additives. Further analyses reveal that Sn^2+^ is bound by carboxylate groups in the carboxymethyl-dextran coating of Fer, thus stabilizing SnF_2_ and boosting the catalytic activity. Notably, Fer in combination with SnF_2_ is exceptionally effective in controlling dental caries *in vivo*, preventing enamel demineralization and cavitation altogether without adverse effects on the host tissues or causing changes in the oral microbiome diversity. The efficacy of SnF_2_ is also enhanced when combined with Fer, showing comparable therapeutic effects at four times lower fluoride concentration. Enamel ultrastructure examination shows that fluoride, iron, and tin are detected in the outer layers of the enamel forming a polyion-rich film, indicating co-delivery onto the tooth surface. Overall, our results reveal a unique therapeutic synergism using approved agents that target complementary biological and physicochemical traits, while providing facile SnF_2_ stabilization, to prevent a widespread oral disease more effectively with reduced fluoride exposure.

## Introduction

Dental caries is the most prevalent and costly biofilm-induced oral disease that causes the destruction of the mineralized tooth tissue^1^. In caries-inducing (cariogenic) biofilms, microorganisms form highly protected biostructures that create acidic pH microenvironments, promoting cariogenic bacteria growth and acid dissolution of tooth-enamel^2, 3^. Despite increased use of fluoride (the mainstay anticaries agent), it remains unresolved and affects 3.1 billion people worldwide, with annual costs exceeding US $290 billion^4, 5^. Even though fluoride is effective in reducing tooth enamel demineralization at acidic pH values^6, 7^, it has limited antibiofilm activity despite inhibitory effects against planktonic bacteria^8.^ Additionally, current modalities, including high-dose fluoride treatments, are insufficient to prevent dental caries in high-risk individuals where pathogenic dental biofilms rapidly accumulate under sugar-rich diets and poor oral hygiene that enables firm bacterial adhesion to teeth^9, 10^. Furthermore, the level of exposure to fluoride that provides strong protection has accompanying risks (e.g., dental fluorosis), especially for children^11, 12, 13^, as fluoride overexposure has detrimental effects^14, 15.^

Ferumoxytol (Fer), an aqueous iron oxide nanoparticle formulation approved by the Food and Drug Administration (FDA) for systemic treatment of iron deficiency, has shown both efficacy and specificity against cariogenic biofilms when used topically, through selective pathogen binding and acidic pH-activation of hydrogen peroxide (H_2_O_2_) via catalytic (peroxidase-like) activity^16, 17^. Although topical applications of Fer can reduce dental caries *in vivo*, it does not interfere physiochemically with enamel demineralization and is unable to entirely prevent the progression of the disease. To improve the efficacy of Fer, combination with fluoride could potentiate the therapeutic effects. We hypothesized that Fer and fluoride could complement each other’s properties, even at lower concentrations, to target the development of dental caries more effectively without increasing fluoride exposure.

**Figure 1:**
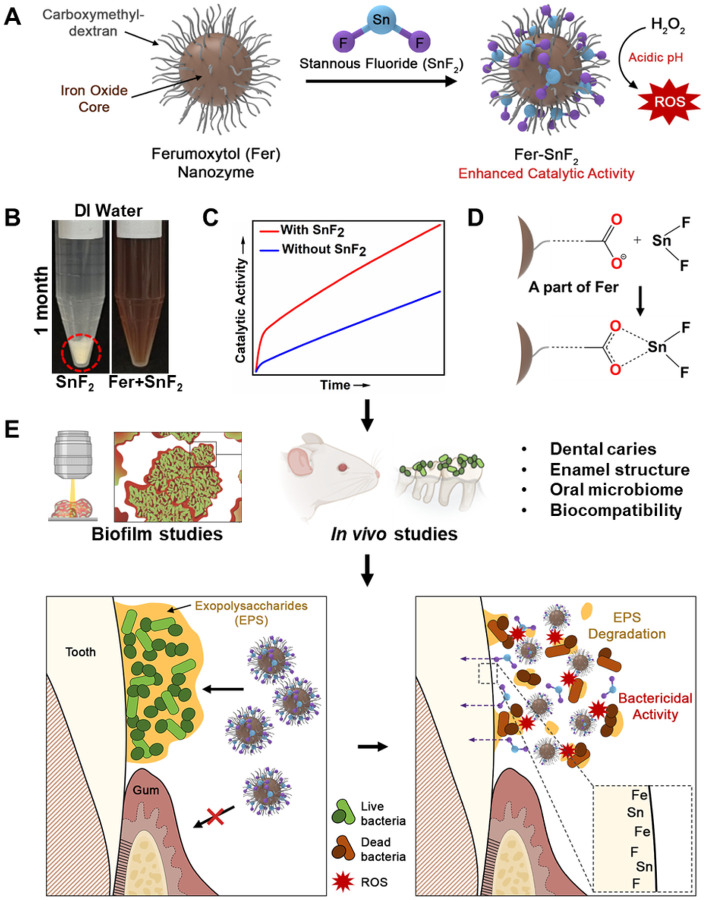
Chemical interactions and therapeutic activity of the combined treatment of Fer and SnF_2_. **(A to C)** Fer chemically interacts with SnF_2_ (A) to enhance stability in aqueous solution without any additives (B) while boosting catalytic activity (C). **(D)** Further biochemical and spectrometry analyses reveal that Sn^2+^ is bound by carboxylate groups in the carboxymethyl-dextran coating of Fer. **(E)** Using laboratory and *in vivo* models, we find synergistic activities to enhance bioactivity against biofilms and caries-protective effects (without increasing fluoride exposure), while co-delivering fluoride, iron, and tin on the outer enamel surface without deleterious effects on oral tissues and the microbiota.

Herein, we evaluated the combination of Fer with two formulations of fluoride widely used in oral health care, sodium fluoride (NaF) and stannous fluoride (SnF_2_). While the combination with NaF did not show improvement, when Fer was combined with SnF_2_ we observed remarkable synergistic effects in vitro and *in vivo*. We found that SnF_2_ was highly stable in aqueous solution when mixed with Fer; the lack of stability of SnF_2_ has been a major limitation in commercial formulations, requiring use of chemical additives^18, 19^. Unexpectedly, the catalytic activity of Fer significantly increased when mixed with SnF_2_, thereby enhancing antimicrobial potency. Further analysis revealed that Sn^2+^ was bound by carboxylate groups in the carboxymethyl-dextran coating of Fer, thereby enhancing the stability of SnF_2_. When tested in a rodent model, we found that Fer in combination with SnF_2_ was exceptionally effective in preventing dental caries (substantially superior to either alone), completely blocking enamel cavitation, an outcome not observed before. Moreover, the anticaries efficacy was achieved at four times lower dosage of SnF_2_. Notably, fluoride, iron, and tin were detected in the outer layers of the enamel, indicating co-delivery to form a caries-protective film *in situ*. Altogether, we developed a combination therapy with unexpected synergistic mechanisms that target the biological (biofilm) and physicochemical (enamel demineralization) traits simultaneously while providing a facile SnF_2_ stabilization and lower dosage strategy against a widespread and costly oral disease, as summarized in Fig. 1.

## Results

### Antibiofilm activity of Fer in combination with SnF_2_ in vitro

Fluoride is widely used as a gold standard anticaries agent, but it does not provide full protection, especially in severe cases where pathogenic biofilms rapidly accumulate. Despite its limited antibiofilm activity, sodium fluoride (NaF) can affect bacterial glycolysis and acid tolerance^20, 21, 22^, whereas stannous fluoride (SnF_2_) provides stronger antibacterial activity imparted by Sn^2+^ ions^23, 24^. First, we tested the antibiofilm activity of both NaF and SnF_2_ (1000 ppm of F, the typical concentration in over-the-counter formulations) and found that SnF_2_ can significantly inhibit *Streptococcus mutans* (*S. mutans*, a cariogenic pathogen) viability and reduce the biomass more effectively than NaF (Fig. S1, A and B). Afterward, we combined NaF or SnF_2_ with Fer (1 mg of Fe/ml, an effective antibiofilm concentration^17^) in the presence of 1% H_2_O_2_ (v/v). Remarkably, the combination of Fer with SnF_2_ has substantially greater antibiofilm activity than the combination of Fer and NaF (Fig. S1, C and D), resulting in no detectable viable bacteria and near complete biomass abrogation. In view of this result, we hypothesized that SnF_2_ might be interacting with Fer for enhanced bioactivity.

**Figure 2:**
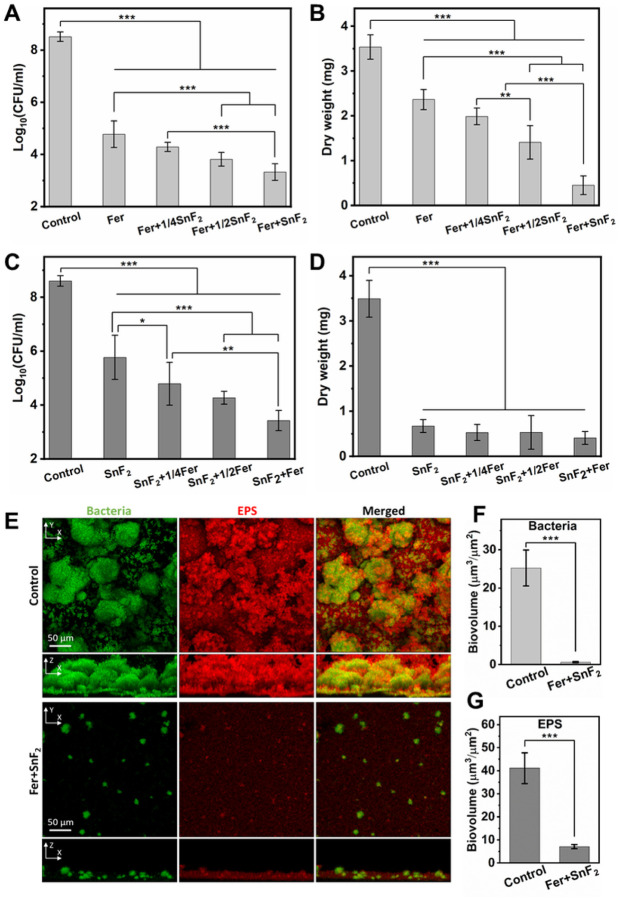
Antibiofilm studies of the combinations of Fer and SnF_2_. **(A** and **B)** The effect of different concentrations of Fer (1 mg of Fe/ml) and SnF_2_ (0–250 ppm of F) on the bacterial viability (A) and the mass of biofilm (B) after 43 h. 1/4SnF_2_, 1/2SnF_2_, and SnF_2_ stand for 62.5 ppm of F, 125 ppm of F, and 250 ppm of F, respectively. **(C** and **D)** The effect of different concentrations of Fer (0–1 mg of Fe/ml) and SnF_2_ (250 ppm of F) on the bacterial viability (C) and the mass of biofilm (D) after 43 h. 1/4Fer, 1/2Fer, and Fer stand for 0.25 mg of Fe/ml, 0.5 mg of Fe/ml, and 1 mg of Fe/ml, respectively. **(E)** Confocal microscopy images of biofilms with or without treatment with Fer (1 mg of Fe/ml) and SnF_2_ (250 ppm of F) after 43 h. Bacterial cells were stained with SYTO 9 (in green), and EPS was labeled with Alexa Fluor 647-dextran conjugate (in red). **(F** and **G)** Quantitative analysis of biovolume of bacterial cells (F) and EPS (G) in the biofilm with or without Fer+SnF_2_ (analyzed using COMSTAT). All biofilms except the control group were treated with H_2_O_2_ (1%, v/v) for 5 min at the end of the experimental period (43 h) before the analysis. The data are presented as mean ± standard deviation. *p < 0.05, **p < 0.01, ***p < 0.001; one-way ANOVA followed by Tukey test.

We then investigated antibiofilm activity using two dose-response studies using varying concentrations of Fer and SnF_2_. Given the potency of combination and high fluoride (1000 ppm of F) concentration, we used the lowest dosage of SnF_2_ (250 ppm of F) known to provide therapeutic effect as upper fluoride dose limit. First, Fer dose (1 mg of Fe/ml) was fixed and mixed with various concentrations of SnF_2_ (0–250 ppm of F), and the number of viable cells and biomass were determined. As expected, Fer displayed a strong antibacterial effect against *S. mutans* biofilm (> 3-log reduction of viable cells; Fig. 2A), while also reducing biomass (Fig. 2B). When Fer was mixed with increasing concentrations of SnF_2_, both the antibacterial activity and the inhibitory effect on the biomass enhanced in a dose-dependent manner, indicating that SnF_2_ can help improve the antibiofilm efficacy of Fer. Next, the Fer concentration was varied (0–1 mg of Fe/ml) at constant SnF_2_ dose (250 ppm of F). When combined with Fer, the antibacterial effect of SnF_2_ increased in a dose-dependent manner, resulting in > 5-log reduction of viable cells compared to control when the concentration of Fer reached 1 mg of Fe/ml. Notably, the combination of Fer and SnF_2_ was at least 2500-fold more effective in killing *S. mutans* cells than SnF_2_ alone (Fig. 2C), suggesting a synergistic effect. We also found that SnF_2_ (250 ppm of F) substantially reduces biomass (Fig. 2D), although inclusion of increasing amounts of Fer did not enhance the bioactivity. The reduction of dry biofilm mass in response to SnF_2_ treatment is likely due to the inhibition of secreted glucosyltransferases that are integral to the production of exopolysaccharides (EPS) by *S. mutans*, as reported by others^25^.

To further assess the antibiofilm activity of the combination of Fer (1 mg of Fe/ml) and SnF_2_ (250 ppm of F), confocal imaging was performed using fluorescent labeling of the bacterial cells and α-glucan EPS. As depicted in Fig. 2E, the control biofilm contained bacterial clusters (in green) spatially arranged with abundant EPS (in red) matrix forming a densely packed structure. In a sharp contrast, the combination of Fer and SnF_2_ impaired the accumulation of the biofilm where only small cell clusters with sparsely distributed EPS were detected. The orthogonal view images revealed that the spatial distribution of bacteria and EPS across the biofilm thickness was substantially compromised in the combination treated biofilm. These findings were further confirmed by quantitative computational analyses, which showed that the combination of Fer and SnF_2_ markedly reduced the biovolume of bacterial cells (Fig. 2F) and EPS (Fig. 2G).

### SnF_2_ stability in combination with Fer

Given the enhanced efficacy of the combination of Fer and SnF_2_, we further investigated the physicochemical properties of this combination. The hydrodynamic diameter of Fer did not change significantly after adding SnF_2_ (Table S1), indicating that SnF_2_ is stable in the solution of Fer. Additionally, we noticed that the zeta potential of Fer (Table S2) became less negative, serving as evidence that Fer interacts with SnF_2_, since coordination of Sn^2+^ by the carboxylate groups is expected to lower the charge density of the carboxymethyl-dextran (CMD) corona. Representative transmission electron microscopy (TEM) images of Fer and Fer + SnF_2_ after 1 h incubation in 0.1 M sodium acetate buffer (pH 4.5) are presented in Fig. S2. Consistent with dynamic light scattering (DLS) data, mixing Fer with SnF_2_ did not seem to affect the size of Fer.

**Figure 3:**
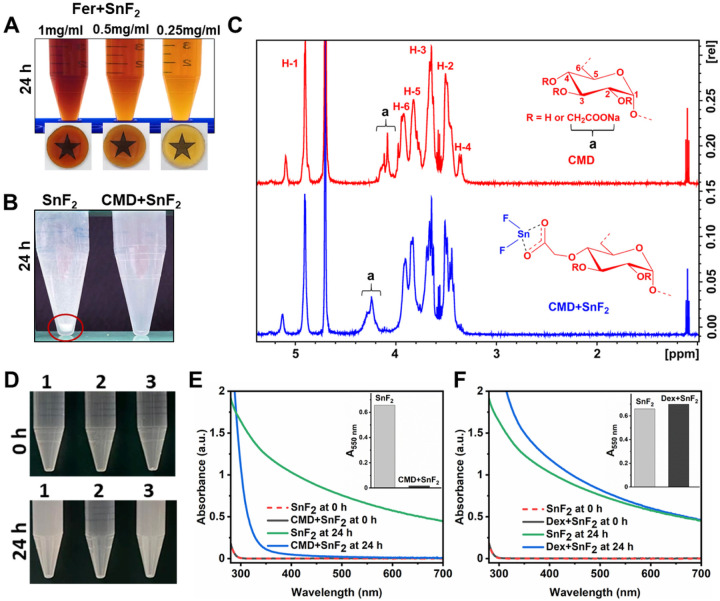
Enhanced stability of SnF_2_ in the presence of Fer. **(A)** Photographs of the combinations of SnF_2_ and Fer at different concentrations of Fer (0.25–1 mg of Fe/ml) at pH 4.5 (0.1 M sodium acetate buffer) after 24 h incubation. **(B)** Photographs of SnF_2_ and the combination of CMD and SnF_2_ at pH 5.5 (0.1 M sodium acetate) after 24 h incubation. The red circle highlights precipitate. **(C)** ^1^H NMR spectra of CMD and CMD+SnF_2_, ‘a’ peaks represent the protons of carboxymethyl groups. **(D)** Photographs of SnF_2_ in different conditions at pH 4.5 (0.1 M sodium acetate buffer). The samples are: 1. SnF_2_ alone, 2. SnF_2_+CMD, and 3. SnF_2_+dextran (Dex). Top: 0 h; bottom: after 24 h. **(E)** UV-visible absorption spectra of SnF_2_ (250 ppm of F) with or without CMD (1 mg/ml) at pH 4.5 (0.1 M sodium acetate buffer) after 0 or 24 h incubation. The inset of (E) shows the absorbance of SnF_2_ and CMD+SnF_2_ at 550 nm after 24 h incubation as a measure of turbidity. **(F)** UV-visible absorption spectra of SnF_2_ (250 ppm of F) with or without Dex (1 mg/ml) at pH 4.5 (0.1 M sodium acetate buffer) after 0 or 24 h incubation. The Inset of (F) shows the absorbance of SnF_2_ and Dex+SnF_2_ at 550 nm after 24 h incubation as a measure of turbidity.

It is noteworthy that SnF_2_ has limited stability in aqueous solutions owing to its high susceptibility to hydrolysis and oxidation^26, 27^ requiring chemical additives (e.g., chelating agents) or anhydrous formulation^19^, which can reduce fluoride bioavailability. We unexpectedly found that SnF_2_ was stable in aqueous solutions containing Fer. To further investigate the stability of SnF_2_ in the presence of Fer, SnF_2_ (250 ppm of F) was mixed with increasing amounts of Fer in 0.1 M sodium acetate buffer at pH 4.5. We observed that the solution containing SnF_2_ mixed with Fer was limpid after 24 h in sodium acetate buffer at pH 4.5 (Fig. 3A), demonstrating that Fer can enhance the stability of SnF_2_.

The enhanced stability of SnF_2_ in the presence of Fer motivated us to investigate their chemical interactions. The core of Fer is coated with carboxymethyl-dextran (CMD)^28^. Thus, we explored whether SnF_2_ can interact with CMD. SnF_2_ alone or mixed with CMD was incubated in 0.1 M sodium acetate buffer (pH 5.5) for 24 h. We observed immediate formation of a precipitate when SnF_2_ was dissolved in sodium acetate buffer, whereas the solution was limpid when mixed with CMD even after 24 h incubation (Fig. 3B). In addition, the mixture of SnF_2_ and CMD was characterized by ^1^H nuclear magnetic resonance (NMR) spectroscopy (Fig. 3C). In the CMD spectrum, the anomeric proton (H1) in the C1 position was identified at 4.9 ppm, and protons (H2-H6) at the C2-C6 positions were detected at 3.2–4.0 ppm. The peak at 4.0–4.2 ppm (denoted as “a”) is attributed to the protons of the carboxymethyl moieties as determined previously^29^. After CMD was mixed with SnF_2_, a clear shift in peak “a” was observed when compared to that of CMD alone. This suggests that Sn^2+^ binds to the carboxymethyl moieties of CMD, which may account for the enhanced stability of SnF_2_ with Fer. Note that similar ^1^H NMR studies of SnF_2_ and Fer are not possible due to the superparamagnetic nature of Fer interfering with ^1^H NMR measurements.

In order to further investigate the effects of CMD on SnF_2_ stability, we compared it with several control materials, i.e. dextran (Dex) (a similar polymer to CMD, but without carboxylic acid groups), as well as citric acid (CA), L-ascorbic acid (AA), and poly(acrylic acid) (PAA), which are all entities that all contain carboxylic acid groups. We found that Dex did not enhance the stability of SnF_2_, whereas each material that contains carboxylic acid groups did enhance stability (Fig. 3, D to F and Fig. S3). The Fer formulation also contains mannitol (Man)^28^, which is an antioxidant^30^. Since antioxidants can prevent the oxidation of SnF_2_^31^, we added SnF_2_ to various amounts of Man (1–10 mg/ml). Surprisingly, we did not observe any noticeable change in the stability of SnF_2_ even with excess amounts of Man (10 mg/ml) (Fig. S4), implying that Man does not have any noticeable effect on enhancing the stability of SnF_2_. All of these findings suggest that Sn^2+^ is bound to carboxylate groups, thereby enhancing the stability of SnF_2_ when mixed with Fer.

### Catalytic activity of Fer in combination with SnF_2_

**Figure 4:**
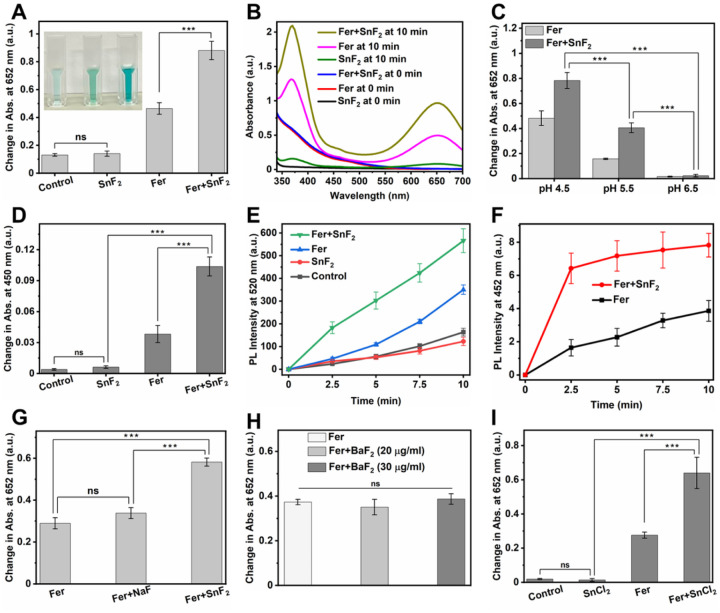
Enhanced catalytic activity of Fer in the presence of SnF_2_. **(A)** Change in the absorption of TMB (chromogenic substrate) at 652 nm in different conditions. Inset: Photographs of TMB incubated with various reagents (left: SnF_2_ alone, middle: Fer alone, and right: Fer+SnF_2_) 10 min after H_2_O_2_ addition. **(B)** UV-visible absorption spectra of TMB in the presence of SnF_2_, Fer, or Fer+SnF_2_ at the times indicated. **(C)** Peroxidase-like activity of Fer and Fer+SnF_2_ at three pH values (4.5, 5.5, and 6.5) as determined by the colorimetric assay using TMB. **(D)** Change in the absorption of OPD at 450 nm in different conditions. The increase in absorption at 450 nm shows ROS production. **(E)** Comparison of change in PL intensities of DCF at 520 nm at various conditions. The increase in PL intensity at 520 nm depicts ROS production. **(F)** The change in PL intensity of 7-hydroxycoumarin at 452 nm as a function of time in the presence Fer with or without SnF_2_. The increase in the PL intensity at 452 shows the generation of •OH. **(G** to **I)** Effect of NaF (G), BaF_2_ (H), and SnCl_2_ (I) on the catalytic activity of Fer in 0.1 M sodium acetate buffer (pH 4.5). The data are presented as mean ± standard deviation. ***p < 0.001; ns, nonsignificant; one-way ANOVA followed by Tukey test.

To explore whether SnF_2_ could influence the catalytic activity of Fer, we used the 3,3′,5,5′-tetramethylbenzidine (TMB) colorimetric assay for peroxidase-like activity following a previously published protocol^32^, with some modifications. TMB is a chromogenic compound that yields a blue color upon oxidation with an absorption peak at 652 nm in the presence of reactive oxygen species (ROS), such as hydroxyl radical (•OH)^33^. As shown in Fig. 4, A and B, SnF_2_ alone did not produce a noticeable amount of ROS. In contrast, the catalytic activity of Fer increased significantly after combining with SnF_2_ as demonstrated by increased colorimetric reaction (Fig. 4, A and B), suggesting that SnF_2_ enhanced the catalytic activity of Fer. Photographs in the inset of Fig. 4A exhibit the color change in each condition (SnF_2_, Fer, and Fer + SnF_2_ from left to right, respectively).

Notably, we found that the enhancement of ROS production in the presence of SnF_2_ is dependent on pH, concentration, and incubation time. The highest catalytic activity was observed at pH 4.5 (Fig. 4C). The greater ROS production at acidic pH conditions (characteristic of pathological conditions associated with dental caries) and the minimal ROS generation close to neutral (physiological) pH suggests a selectivity toward pathogenic bacteria. Surprisingly, very small amounts of SnF_2_ are adequate for enhancing the catalytic activity of Fer (Fig. S5), whereby more ROS can be detected within 10 min incubation (Fig. S6A) gradually increasing to reach the highest level at 6 h (Fig. S6B), which was maintained for prolonged period.

To further confirm the enhancement of the peroxidase-like activity of the Fer in the presence of SnF_2_, we employed a multi-pronged approach. First, we used OPD, a colorless substrate, which yields an oxidized product with a characteristic yellow color when reacting with ROS with an absorption peak at 450 nm^34^. As expected, the catalytic activity of Fer increased markedly after adding SnF_2_ as compared to Fer alone and SnF_2_ alone (Fig. 4D). We also measured ROS production via photoluminescence (PL) method using DCFH-DA as a ROS tracking indicator. DCFH-DA (a nonfluorescent molecule) yields a fluorescent molecule DCF in the presence of ROS^35^. As depicted in Fig. 4E, the PL intensity increased to a greater extent after combining Fer with SnF_2_. Then, we measured the amount of hydroxyl radical (•OH) using coumarin as a photoluminescent probe molecule^36, 37^. As seen in Fig. 4F, Fer and SnF_2_ in combination generated significantly more •OH than Fer alone, further demonstrating that SnF_2_ enhanced the catalytic activity of Fer. In contrast, SnF_2_ alone did not produce a noticeable amount of •OH (Fig. S7), consistent with its lack of catalytic activity.

Next, we investigated whether the augmented catalytic activity arises from different fluoride or stannous salts. We replaced SnF_2_ with NaF, a commonly used fluoride salt in oral care formulations, or barium fluoride (BaF_2_), another fluoride salt with a divalent cation of comparable size to Sn^2+^. We found that neither NaF nor BaF_2_ increased the catalytic activity of Fer noticeably (Fig. 4, G and H), suggesting that F^−^ may not play a crucial role in enhancing the ROS production performance of Fer. Conversely, we used SnCl_2_ to evaluate whether Sn ions play a role in strengthening the ROS generation capability of Fer. We found SnCl_2_ enhanced the catalytic activity of Fer (Fig. 4I), indicating that Sn ions may be playing a dominant role in increasing the catalytic performance of Fer. Taken together, these findings support that SnF_2_ can boost the catalytic ability of Fer, indicating that Fer and SnF_2_ combination is an effective ROS-generating therapy that can target biofilms under pathological (acidic) conditions.

We examined whether Fer released iron ions when combined with SnF_2_ using inductively coupled plasma optical emission spectroscopy (ICP-OES). As depicted in Fig. S8A, the presence of SnF_2_ slightly increased iron ions release from Fer at acidic pH (4.5). It is noteworthy that the amount of leached irons from Fer + SnF_2_ formulation at circumneutral pH is negligible (Fig. S8B). Conversely, the iron leached from Fer + SnF_2_ at acidic pH values could provide an added benefit. Iron ions have shown cariostatic effects as they can precipitate on the surface of enamel and promote the adsorption of phosphate and calcium ions, thereby reducing enamel demineralization^38, 39^.

Altogether, the increased stability of SnF_2_ in aqueous solutions is mediated at least in part via interactions with CMD, which may be important for both fluoride bioavailability and fluoride delivery. Unexpectedly, the presence of SnF_2_ boosts the ROS generation capability of Fer at acidic pH, thus enhancing antibiofilm efficacy under pathological condition. This synergistic Fer and SnF_2_ combination provide a potent yet pH-dependent ROS-based therapy with enhanced antimicrobial fluoride stability that could prevent the onset of dental caries *in vivo*.

### Biocompatibility of Fer+SnF_2_ in vitro

To examine whether this combination treatment is viable for use *in vivo*, the cytotoxicity of the combination of Fer and SnF_2_ was assessed in human gingival keratinocytes (HGK) using MTS assay. The cells were incubated with the combination of Fer (1 mg of Fe/ml) and SnF_2_ (250 ppm of F) for 10 min, followed by 24 h incubation with fresh cell culture media. We found that the combined treatment of Fer and SnF_2_ had no adverse effect on cell viability (Fig. S9).

### Impact of Fer/SnF_2_ on caries development and on enamel surface in vivo

**Figure 5:**
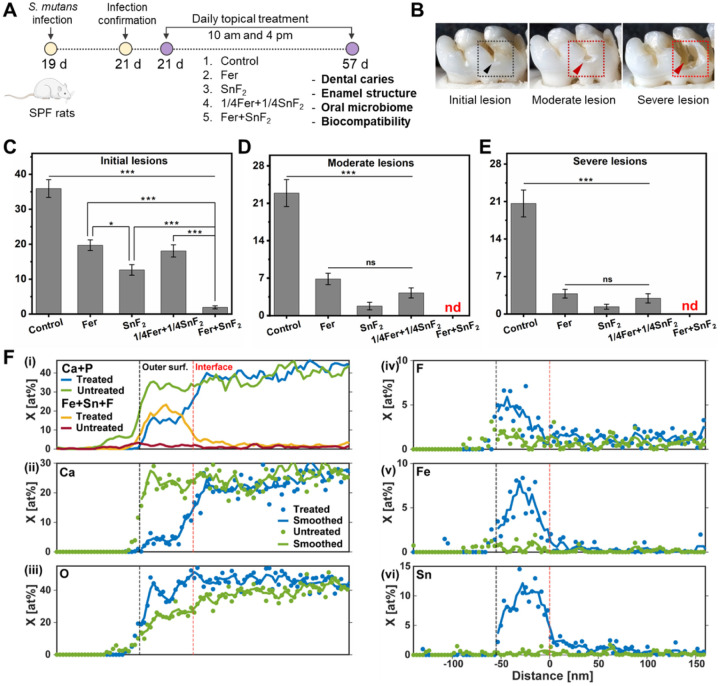
Therapeutic efficacy of the combination of Fer and SnF_2_ against dental caries and elemental composition of the surface of the treated tooth enamel *in vivo*. **(A)** Experimental design of *in vivo* study. **(B)** An illustration showing initial lesion (enamel affected), moderate lesion (dentin affected), and severe lesion (full cavitation). **(C** to **E)** Caries scores recorded from smooth surface. The caries scores were recorded according to Larson’s modification of Keyes’ scoring system for stages and extent of carious lesion severity. The data are presented as mean ± standard error of the mean. *p < 0.05, ***p < 0.001; ns, nonsignificant; find, nondetectable; one-way ANOVA followed by Tukey test. **(F)** Plots of sums of mole fractions of Ca and P, and of Fe, Sn, F (i), and plots of mole fractions of Ca (ii), O (iii), F (iv), Fe (v), and Sn (vi) vs. distance in the direction normal to the EES for M1 rat molars from rates treated with Fer+SnF_2_ and untreated controls. Profiles were manually aligned on the outer surface, and that the distance axis is referenced to the approximate position of the interface between the Fe/Sn/F-rich layer and the underlying enamel of the treated sample.

Topical applications of Fer and SnF_2_ *in vivo* were assessed using a rodent model that mimics the characteristics of severe human caries^40^, including sugar-rich diet and the development of surface zones^41^. Rat pups were infected with *S. mutans* (oral bacterial pathogen) and fed a sugar-rich diet (Fig. 5A). In this model, as depicted in Fig. 5B, tooth enamel progressively develops caries lesions (analogous to those observed in humans), proceeding from initial areas of demineralization to severe lesions characterized by enamel structure damage and cavitation. The test agents were topically applied twice daily with 1 min exposure time (Fig. 5A) to mimic the clinical use of a mouthwash. After the experimental period, the incidence and severity of caries lesions were evaluated. We also included a reduced concentration of the combination of Fer (0.25 mg of Fe/ml) and SnF_2_ (62.5 ppm of F), since the lower amounts were capable of significantly killing the bacteria (p < 0.001) and reducing biomass (p < 0.001) compared to control *in vitro* (Fig. S10).

Quantitative caries scoring analyses revealed that the treatment of Fer in combination with SnF_2_ was exceptionally effective in preventing caries development with higher efficacy than either alone (*p* < 0.001) (Fig. 5C). It nearly abrogated caries initiation and completely blocked further caries lesions development, thus preventing the onset of cavitation altogether (Fig. 5, D and E). The efficacy of the lower dosage of Fer and SnF_2_ treatment was significantly greater than the control group (*p* < 0.001), and as effective as Fer (1 mg of Fe/ml) or SnF_2_ (250 ppm of F) treatment alone. This demonstrates that the combination of Fer and SnF_2_ has a synergistic effect for efficient biofilm treatment *in vivo*.

To determine the impact of treatment on the elemental composition of the enamel surface, lamellae oriented normal to the external enamel surface (EES) of rat mandibular first molars (M1) were lifted out using a conventional focused ion beam (FIB) technique (Fig. S11). Line profiles normal to the EES were determined by scanning transmission electron microscopy (STEM) with energy dispersive spectroscopy (STEM-EDS) (Fig. 5F) and aligned to the outer surface (see Methods). Comparing M1 from Fer + SnF_2_ and control groups (Fig. 5F(i)), we find that the combined mole fractions of Fe, Sn, and F are substantially elevated, and the sum of the mole fractions Ca and P correspondingly reduced, in a thin film at the surface. Preliminary analyses revealed that the thickness of this film varies from ~ 50 to greater than 300 nm. Inspection of single element profiles from a 50 nm-thick film (Fig. 5F(ii to vi)) reveals that while the calcium mole fraction is reduced from ~ 25 at% to less than 5 at% in this layer, the mole fraction of oxygen remains at the same level as in the underlying enamel (Fig. 5F(iii)). Sn reaches slightly more than 10 at% (Fig. 5F(vi)), while Fe is closer to 7 at% (Fig. 5F(v)). While the profiles of the latter show broad maxima in the center of the layer, F levels appear highest at the outer surface at ~ 5 at% and decline to ~ 1 at% at the interface (Fig. 5F(iv)). The presence of a Fe/Sn/F-rich layer was confirmed on a separately prepared sample from the same, treated rat molar using STEM with electron energy loss spectroscopy (STEM-EELS) (Fig. S12). Furthermore, the presence of Fe, Sn, and F at the surface of treated teeth, and the absence of these ions on vehicle-treated controls was confirmed using X-ray photoelectron spectroscopy (XPS, Fig. S13).

In high-resolution TEM (HRTEM) images, the Fe/Sn-rich layer was apparent as a slightly darker band between the enamel and the protective layers of FIB-deposited carbon and FIB-deposited Pt/C (Fig. S14). Lattice fringes were readily apparent in enamel (Fig. S14A), and Fast Fourier transform (FFT) images and radial integrals (Fig. S14, B and E) revealed sharp features consistent with the {002}, $\left\{3\;\;\bar{2}\;\;1\right\}$, and $\left\{3\;\;\bar{3}\;\;0\right\}$ sets of planes of crystalline hydroxylapatite. The Fe/Sn-rich layer did not display lattice fringes, and FFT images only showed diffuse scattering with a broad maximum at ~ 0.33 nm^−1^ (Fig. S14, C and F), consistent with an amorphous oxide layer.

Taken together, there is strong evidence that there is a layer comprised of Fe and Sn with varying amounts of fluoride present at the surface of the teeth of animals treated with SnF_2_ + Fer. The presence of Ca and P in this layer may indicate co-precipitation during its formation. Gradients of Fe, Sn, and F at the interface between the film and the underlying enamel suggest that these ions diffuse into enamel, but this remains to be confirmed.

### Effect of Fer/SnF_2_ on host microbiota and oral tissues in vivo

**Figure 6:**
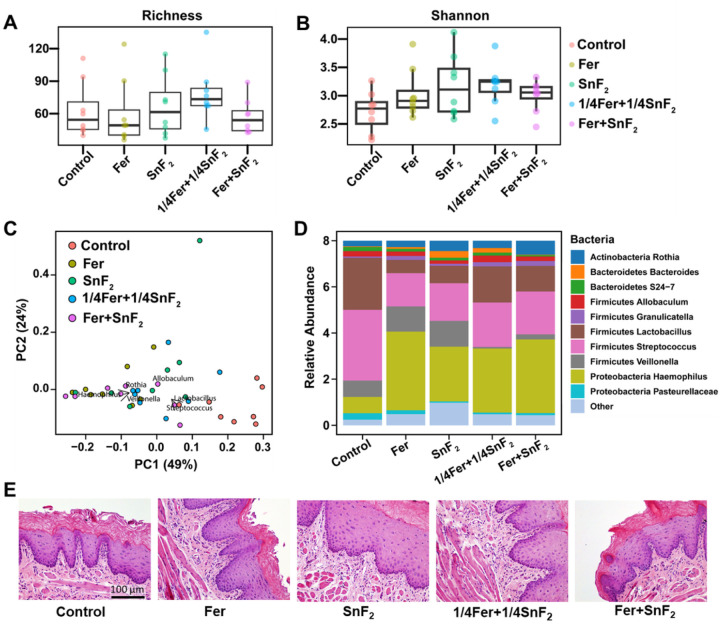
Effects of Fer and SnF_2_ on oral microbiome and gingival tissue *in vivo* post-treatment. **(A** and **B)** Alpha diversity measured by Richness and Shannon indexes shows no significant differences among groups. **(C)** Principal coordinate analysis (PCoA) using weighted UniFrac distances reveal that the Fer+SnF_2_ group has a similar composition and the lowest distances between samples. **(D)** The bar plot shows the main bacterial genera found across all samples, distributed by treatment groups. **(E)** Histology of the gingival tissue with treatments noted. Fer, 1/4Fer, SnF_2_, and 1/4SnF_2_ stand for 1 mg of Fe/ml, 0.25 mg of Fe/ml, 250 ppm of F, and 62.5 ppm of F, respectively.

The effects of Fer and SnF_2_ on oral microbiota and surrounding soft tissues were also evaluated to assess the impact on oral microbiome diversity and oral tissue toxicity. All treatment groups showed no significant differences in alpha diversity among each group (Fig. 6, A and B, p > 0.05, Willcox test). Furthermore, weighted UniFrac distances analyzed of principal coordinate analysis (PCoA) by treatment groups revealed that Fer and SnF_2_ treatment group has a similar composition with the lowest dispersion (Fig. 6C, green dots), indicating no deleterious effects on the oral microbiota diversity (*p* > 0.05, PERMANOVA). Notably, microbiome data revealed a higher abundance of acidogenic bacterial genera such as *Streptococcus* and *Lactobacillus* in the control group, whereas they decreased in all the treatments (Fig. 6, C and D). *Veillonella* is known to consume acids produced by other acidogenic oral bacteria to grow and survive. *Veillonella* is especially reduced in the combined treatment groups, i.e. 1/4Fer + 1/4SnF_2_ and Fer + SnF_2_ (Fig. 6, C and D), indicating reduced acidogenic environment. In contrast, commensal genera related to oral health such as *Haemophilus* and *Rothia* were increased in treatment groups. Altogether, the microbiome data indicates that bacterial diversity is not affected as a community (Fig. 6D), but specific bacteria associated with pathogenic environment are reduced by the combination treatment.

Histopathological analysis of gingival tissues revealed no indication of an acute inflammatory response, cytotoxicity, necrosis, or any changes in vascularization or proliferation, suggesting biocompatibility of Fer and SnF_2_ treatment (Fig. 6E), consistent with *in vitro* data. Collectively, the data show that the combination was substantially more potent than either alone, whereas a lower concentration of agents in combination was as effective as each alone at full strength, indicating a synergistic effect between Fer and SnF_2_. In addition, the treatments did not disrupt the ecological balance of the oral microbiota or cause deleterious effects on the surrounding host tissues, indicating high precision for targeting cariogenic plaque-biofilms and preventing disease progression *in vivo*.

## Discussion

In summary, we unexpectedly found a remarkable synergy between ferumoxytol (Fer) nanozymes and stannous fluoride (SnF_2_) in potentiating antibiofilm and anticaries efficacy, which is particularly relevant given that current treatments are insufficient for controlling biofilm and preventing demineralization simultaneously in high-risk populations prone to disease. The combination treatment is far more effective than either alone and completely halts the progression of caries lesions and cavitation in a rodent model, without adverse effects on the surrounding host tissues or on the oral microbiota diversity *in vivo*.

Notably, we observed initial enamel lesions and eventually cavity formation when treated with SnF_2_ or Fer alone, indicating that sufficient acid is still generated to attack enamel. In sharp contrast, early lesions were seldom seen when treated with SnF_2_ in combination with Fer. Furthermore, comparable therapeutic effects were achieved even at 4 times lower fluoride concentration (62.5 ppm of F) when mixed with Fer, demonstrating the possibility of a therapy that uses very low doses (typical amounts in oral formulations ranges from 1000 to 1500 ppm of F). Such therapeutic synergy has not been observed previously in this animal model that mimics severe disease. Further analyses revealed that the improved effects achieved with the combination system can be attributed to three factors: (i) stabilization of SnF_2_ through tin-carboxymethyl interactions, (ii) significant enhancement of the catalytic activity of Fer, and (iii) formation of a Fe/Sn/F-rich film at the outer surface of the tooth enamel. These properties acting in concert can potentiate antibiofilm activity and enhance enamel resistance against demineralization, while also displaying therapeutic effect at lower dosages.

It is well known that SnF_2_ undergoes oxidation and hydrolysis in aqueous solutions. Therefore, maintaining the stability and efficacy of SnF_2_ has been a ubiquitous challenge since the inception of the use of SnF_2_ in the 1950s. Several formulations have been developed to enhance the stability of SnF_2_ in oral care products, including (i) removal of water from the formula or use of low water content, (ii) addition of extra stannous salts, such as SnCl_2_, as a sacrificial source of stannous ions, and (iii) addition of complexing agents, such as zinc phosphate^19^. However, the currently available methodologies have some limitations, including interactions of stannous complexes with other ligands present in toothpaste that affect the therapeutic efficacy of SnF_2_^42^. Herein, we offer an alternate and facile approach of stabilizing SnF_2_ in aqueous solutions with additional therapeutic benefits. Specifically, we show (i) Fer can stabilize SnF_2_ via simple mixing without using any excipient ingredients while (ii) enhancing anticaries effect of SnF_2_ when mixed with Fer. We find that Sn binding through the carboxylate groups of the Fer nanozyme formulation contributes to the stabilization of SnF_2_ and also plays a role in enhancing the catalytic activity of Fer.

Our data indicate that Sn^2+^ rather than F^−^ is responsible for increasing the peroxidase-like activity of Fer. It is possible that Sn^2+^ in close proximity to the nanozyme core could efficiently accelerate the Fe^2+^/Fe^3+^ redox cycles, while Sn-bound in the vicinity could serve as electron donors, resulting in electron transfer between Sn and Fe, thereby increasing ROS production. However, further studies are required to understand the exact mechanisms for catalytic enhancement in this system. Notably, the enhancement of catalytic activity was more pronounced at acidic pH value (4.5), typically found in cariogenic biofilms, whereas minimal ROS was generated close to neutral (physiological) pH, providing high selectivity and antibiofilm activity. Taken together, it provides targeted activity under pathological conditions and operates at acidic pH values at which the anticaries action of SnF_2_ is most effective^43^.

In addition to high antibiofilm specificity and efficacy, the formation of an outer layer film containing Fe, Sn, and F can provide a ‘protective shield’ against enamel acid demineralization. Fluoride acts by inhibiting mineral loss at the crystal surface and enhancing the rebuilding or remineralization of calcium and phosphate in a form more resistant to subsequent acid attacks^44^. The presence of a coating of metal-rich surface precipitate or a metal-rich surface layer can make enamel more acid-resistant^45, 46^. We found a film at the tooth surface that contains both Fe/Sn and F, and also variable amounts of calcium and phosphate. Calcium and phosphates contribute a protective role in preventing enamel demineralization by modulating physicochemical equilibrium and forming CaF_2_ with fluoride that reduces acid solubility while promoting remineralization^47^. To the best of our knowledge, the formation of Fe/Sn/F polyion film has not been described previously and potentially a novel mechanism for caries prevention.

Despite promising results, there are some limitations, but also opportunities for further research. Although our preliminary study suggests that carboxylates play an integral role in enhancing the stability of SnF_2_, additional analyses are needed to understand the physicochemical interactions between SnF_2_ and Fer as well as the long-term stability of the complexes and the oxidation state of Sn in the complexes. Additional studies are required to elucidate the exact mechanisms by which ROS generation is enhanced by SnF_2_. Further analyses on how the metal ion-fluoride film is formed may reveal additional insights on the enamel remineralization process. Additionally, full toxicity studies are needed to determine the long-term effects of daily use of Fer and SnF_2,_ whereas optimization of the concentrations of Fer, SnF_2_, and H_2_O_2_ may be required for clinical translation and product development. Nevertheless, our data reveal that Fer and SnF_2_ potentiate the therapeutic activity through unexpected synergistic mechanisms that target both the biological (biofilm) and physicochemical (enamel demineralization) traits of dental caries simultaneously.

This simple yet effective combination therapy with fluoride co-delivery could advance current anticaries treatment while leading to the development of ROS-based modalities for other biofilm-related diseases. The search for new modalities encompasses novel compounds, where further development involves a lengthy and costly process and regulatory approval. The findings that an off-the-shelf iron oxide nanoparticle formulation has a potent topical effect at a fraction (< 0.2%) of the approved systemic dosage together with low dose of SnF_2_ that operates through complementary mechanisms of action can facilitate its path to clinical translation. This approach could be targeted for high-risk individuals prone to cariogenic biofilm accumulation without increasing the risk of fluoride overexposure. It is noteworthy that patients with severe childhood tooth decay is often linked with iron deficiency anemia^38, 48, 49, 50^. The possibility that two major global health problems, i.e., tooth decay and anemia^50, 51^, could be treated by using Fer and SnF_2_ opens a feasible opportunity to include the combination therapy in clinical trials for caries prevention tailored to high-risk patients with iron-deficiency anemia.

## Methods

### *In vitro* biofilm model and quantitative analysis

Biofilms were formed using the saliva-coated hydroxyapatite disc (sHA) model as described elsewhere^17, 32, 52^. *S. mutans* UA159, a proven virulent and well-characterized cariogenic pathogen, was grown in ultra-filtered (10 kDa, cutoff; Millipore, Billerica, MA) tryptone-yeast extract (UFTYE) broth at 37°C and 5% CO_2_ to mid-exponential phase. Briefly, HA discs (surface area of 2.7 ± 0.2 cm^2^; Clarkson Chromatography Inc., South Williamsport, PA) were vertically suspended in 24-well plates using a custom-made wire disc holder and coated with filter-sterilized human saliva for 1 h at 37 °C. Each sHA disc was inoculated with ~ 2 × 10^5^ CFU of *S. mutans* per ml in UFTYE containing 1% sucrose at 37°C and 5% CO_2_. Topical treatment of Fer and SnF_2_ or vehicle control was performed for 10 min at 0, 6, 19, and 29 h. The culture medium was changed twice daily (at 19 h and 29 h). At the end of the experimental period (43 h), the biofilms were placed in 2.8 ml of H_2_O_2_ (1%, v/v) for 5 min. After H_2_O_2_ exposure, the biofilms were removed and homogenized via bath sonication followed by probe sonication (at an output of 7 W for 30 s). The homogenized suspension was serially diluted and plated onto blood agar plates using an automated EddyJet Spiral Plater (IUL, SA, Barcelona, Spain). The number of viable cells in each biofilm were calculated by counting CFU. The remaining suspension was centrifuged at 5500 g for 10 min. Finally, the resulting cell pellets were then washed, oven-dried, and weighed. SnF_2_ and NaF treatment groups were performed according to the same procedure.

To visualize the biomass reduction and EPS degradation, SYTO 9 (485/498 nm; Molecular Probes) was used for labeling bacteria and Alexa Fluor 647-dextran conjugate (647/668 nm; Molecular Probes) was used for labeling insoluble EPS. The 3D biofilm architecture was acquired using Zeiss LSM 800 with a 20x (numerical aperture = 1.0) water immersion objective. The biofilms were sequentially scanned using diode lasers (488 and 640 nm), and the fluorescence emitted was collected with GaAsP or multialkali PMT detector (475–525 nm for SYTO 9 and 645–680 nm for Alexa Fluor 647-dextran conjugates, respectively). ImageJ software was used for biofilm visualization and quantification.

### Characterization of Fer & SnF_2_

Fer (100 μg of Fe/ml) and Fer (100 μg of Fe/ml) + SnF_2_ (100 μg/ml) prepared in DI water were used for determining hydrodynamic diameter and zeta potential. The measurements were carried out using a Nano-ZS 90 (Malvern Instrument, Malvern, UK) at indicated time points. TEM was performed using a Tecnai T12 (FEI Tecnai) electron microscope at 100 kV. In brief, solutions of Fer and Fer + SnF_2_ were prepared in 0.1 M sodium acetate buffer (pH 4.5) and incubated for 1 h. After that, 5 μl of the solution of Fer or Fer + SnF_2_ was dropped onto a TEM grid, and the liquid was dried before microscopy was conducted. ^1^H NMR spectroscopic data of CMD with or without SnF_2_ were recorded using a Bruker DMX 500, equipped with a z-gradient amplifier and 5 mm DUAL (1H/13C) z-gradient probe head, in D_2_O. UV-visible absorption spectra were recorded using a Genesys 150 UV − visible spectrophotometer (Thermo Scientific, Waltham, MA).

### ROS measurement using 3,3’,5,5’-tetramethylbenzidine (TMB) assay

The catalytic activity of Fer + SnF_2_ was investigated by a colorimetric assay using TMB (Sigma-Aldrich) as a probe, which generates a blue color after reacting with ROS^33^. Briefly, the stock solution of TMB was made in DMF (25 mg/ml). Fer (0.5 mg of Fe/ml) and SnF_2_ (0.5 mg/ml) were incubated (separately or combined) at room temperature in 0.1 M of sodium acetate buffer (pH 4.5) for 1 h. Afterward, 40 μl of the testing sample (Fer, SnF_2_, or Fer + SnF_2_) and 4 μl of TMB (100 μg) were added into 922 μl of 0.1 M sodium acetate buffer (pH 4.5), mixed by pipette and absorbance was recorded at 652 nm. Then, 34 μl of H_2_O_2_ (1%, v/v) was added. After 10 min additional incubation in the dark, catalytic activities were monitored at 652 nm. For the control, 40 μl of the buffer solution was taken instead of the testing sample. The effect of pH on the catalytic activity of Fer + SnF_2_ was determined at three different pH values (4.5, 5.5, and 6.5) as described above.

To probe the effect of sodium fluoride (NaF, Sigma-Aldrich) on the catalytic activity of Fer, Fer (0.5 mg of Fe/ml) and NaF (0.5 mg/ml) were incubated (separately or combined) at room temperature for 1 h in 0.1 M sodium acetate buffer (pH 4.5). Subsequently, 40 μl of the testing sample (Fer or Fer + NaF) and 4 μl of TMB (100 μg) were added into 922 μl of 0.1 M sodium acetate buffer (pH 4.5) and then mixed via pipetting, and absorbance was recorded at 652 nm. Afterward, 34 μl of H_2_O_2_ (1%, v/v) was added. Finally, the absorbance of TMB was monitored at 652 nm after 5 min incubation. In a similar way, the effect of barium fluoride (BaF_2_) (final concentration 20 or 30 μg/ml, Sigma-Aldrich) and stannous chloride (SnCl_2_) (final concentration 20 μg/ml, Sigma-Aldrich) on the catalytic activity of Fer was also investigated. The effect of incubation time on the catalytic activity was investigated after incubating Fer and SnF_2_ for a predetermined time as described above. All the reactions were investigated using the Genesys 150 UV − visible spectrophotometer.

### Investigation of ROS generation using o-phenylenediamine (OPD)

The enhancement of the catalytic activity of Fer in the presence of SnF_2_ was further verified by employing OPD (Sigma-Aldrich) as a ROS tracking agent^34^. Briefly, the stock solution of the combination of Fer (0.5 mg of Fe/ml) and SnF_2_ (0.5 mg/ml) was incubated for 1 h in 0.1 M sodium acetate buffer (pH 4.5) at room temperature. Afterward, 40 μl of the mixture of Fer (20 μg of Fe) and SnF_2_ (20 μg) and 4 μl of OPD (100 μg) were added into 922 μl of 0.1 M sodium acetate buffer (pH 4.5) and then mixed via pipetting and absorbance was recorded at 450 nm. After adding 34 μl of H_2_O_2_ (1%, v/v), the mixture was further incubated for 1 min, and the absorbance was recorded at 450 nm.

### ROS study using 2 ,7 -dichlorofluorescin diacetate (DCFH-DA) probe

In order to further support the enhancement of the catalytic activity of Fer in the presence of SnF_2_, we used photoluminescence (PL) method using DCFH-DA (Sigma-Aldrich) as a ROS probing agent^35^. First, stock solutions of Fer (0.5 mg of Fe/ml) with or without SnF_2_ (0.5 mg/ml) were incubated in 0.1 M sodium acetate buffer (pH 4.5) for 1 h at room temperature. Afterward, the working solution (final volume 2 mL) containing DCFH-DA (30 μM) and Fer (20 μg of Fe/ml) with or without SnF_2_ (20 μg/ml) was prepared in 0.1 M sodium acetate buffer (pH 4.5). Subsequently, PL intensity was recorded at 520 nm with an excitation wavelength of 505 nm. H_2_O_2_ (1%, v/v) was then mixed to the reaction mixture to initiate the reaction, and the PL intensity was recorded at 520 nm at different incubation times with the excitation wavelength of 505 nm. For the control, vehicle was used.

### Comparison of hydroxyl radical (•OH) production

•OH generated by Fer and Fer + SnF_2_ in 0.1 M sodium acetate buffer (pH 4.5) was analyzed by a PL technique using coumarin (Sigma-Aldrich) as a •OH trapping molecule^36, 37^. First, stock solutions of Fer(0.5 mg of Fe/ml) with or without SnF_2_ (0.5 mg/ml) were incubated in 0.1 M sodium acetate buffer (pH4.5) for 1 h at room temperature. Afterward, Fer (20 μg of Fe/ml) with or without SnF_2_ (20 μg/ml) was mixed with coumarin (0.1 mM) in a 10 mm path length cuvette, and then H_2_O_2_ (1%, v/v) was added to the reaction mixture to initiate the reaction. The PL intensity was recorded at 452 nm at different incubation times with an excitation wavelength of 332 nm. Vehicle was used as the control.

### Iron release study

The release of soluble iron from Fer, in the presence and absence of SnF_2_, was investigated using inductively coupled plasma optical emission spectroscopy (ICP-OES, Spectro Genesis). Briefly, 10 ml of Fer (0.5 mg of Fe/ml) was incubated with or without SnF_2_ (0.5 mg/ml) for 1 h in 0.1 M sodium acetate buffer (pH 4.5, 5.5, or 6.5) at room temperature. Afterward, free iron ions and intact nanoparticles were separated by centrifugation using ultrafiltration tubes (3 kDa, MWCO). The pellet was then resuspended in the same volume using 0.1 M sodium acetate buffer. Subsequently, the filtrate and resuspend pellet were digested in nitric acid and finally diluted with DI water before analysis by ICP-OES.

### Toxicity study of the combined treatment of Fer and SnF_2_ in human gingival keratinocytes (HGK)

The *in vitro* biocompatibility of the combination of Fer and SnF_2_ was investigated in HGK cells using an MTS [(3-(4,5- dimethylthiazol-2-yl)-5-(3-carboxymethoxyphenyl)-2-(4-sulfophenyl)-2H-tetrazolium)] assay (CellTiter 96 cell proliferation assay kit; Promega, WI, USA). HGK cells were kindly provided by the laboratory of Dana T. Graves (School of Dental Medicine, University of Pennsylvania) and were cultured in keratinocyte growth medium (Lonza, USA). To determine the cytotoxicity, HGK cells were seeded in 96-well plates at a density of 10^4^ cells per well. Cells were then incubated at 37°C in a humidified 5% CO_2_ atmosphere in a cell incubator for 24 h. Afterward, old media was replaced with 100 μl of fresh media with or without Fer (1 mg of Fe/ml) and SnF_2_ (250 ppm of F), or either alone, and incubated for 10 min.

After that, the media was removed, the cells were washed twice with sterile phosphate buffered saline (PBS) and 100 μl of fresh complete cell culture media was added to each well. After 24 h incubation, the cell culture media was removed, and 20 μl of MTS reagent and 100 μl of media were added to each well. After 3 h additional incubation under standard cell culture conditions, the absorbance was recorded at 490 nm using a microplate reader. The cell viability was calculated using the following formula:

$$\text{Cellviability}=\frac{{\text{A}}_{\text{490}}^{\text{treated}}}{{\text{A}}_{\text{490}}^{\text{untreated}}}\times 100\%$$

### *In vivo* efficacy of Fer in combination with SnF_2_

*In vivo* efficacy was assessed using a well-established rodent model of dental caries, as reported previously^40, 53^. In brief, 15 days-old specific pathogen free Sprague-Dawley rat pups were purchased with their dams from Harlan Laboratories (Madison, WI, USA). Upon arrival, animals were screened for *S. mutans* by plating oral swabs on mitis salivarius agar plus bacitracin (MSB). Then, the animals were orally infected with *S. mutans* UA159, and their infections were confirmed at 21 days via oral swabbing. To simulate a clinical scenario, a topical treatment regimen was used that consisted of a short exposure (30 s) to the agent, followed by another short exposure (30 s) to H_2_O_2_ (1%, v/v) (or buffer). All infected pups were randomly placed into five treatment groups, and their teeth were treated twice daily. The treatment groups included: (1) control (0.1 M sodium acetate buffer, pH 4.5), (2) Fer only (1 mg of Fe/ml),(3) SnF_2_ only (250 ppm of F), (4) 1/4 Fer + 1/4SnF_2_ (0.25 mg of Fe/ml and 62.5 ppm of F) and (5) Fer + SnF_2_ (1 mg of Fe/ml and 250 ppm of F). Each group was provided the National Institutes of Health cariogenic diet 2000 (TestDiet, St. Louis, MO) and 5% sucrose water ad libitum. The experiment proceeded for 5 weeks, and their physical appearance was recorded daily. At the end of the experimental period, all animals were sacrificed, and their jaws were surgically removed and aseptically dissected, followed by sonication to recover total oral microbiota as reported previously^54^. All of the jaws were defleshed, and the teeth were prepared for caries scoring based on Larson’s modification of Keyes’ system^40^. Determination of the caries score of the jaws was performed by a calibrated examiner who was blinded for the study by using codified samples. Enamel surfaces were analyzed as described below. Moreover, the gingival tissues were collected for hematoxylin and eosin (H&E) staining for histopathological analysis by an oral pathologist at Penn Oral Pathology. This research was reviewed and approved by the University of Pennsylvania Institutional Animal Care and Use Committee (IACUC #805529).

### Preparation of enamel samples for scanning transmission electron microscopy (STEM)

We identified the most promising location for focused ion beam (FIB) lift-out as the middle cusp of the buccal side by assessing curvature and roughness using synchrotron micro-computed tomography reconstructions of whole molars (mandibular) and 3D measuring laser confocal microscopy (Olympus LEXT OLS5000 equipped with a laser operating at a wavelength of 405 nm). Whole air-dried M1 molars were attached, with the buccal side facing up, to a scanning electron microscopy (SEM) stub with carbon and copper tape (Electron Microscopy Sciences). Specimens were then coated with carbon (~ 10 nm, Denton Desk deposition system). The surface of the middle cusp of the buccal side of the tooth was then investigated in detail for microscopic surface roughness, using electron beam imaging at a high tilt angle (52°). Lamellae were lifted out directly from the surface of the tooth in areas that were sufficiently flat (⪅ 500 nm height modulation), using a dual-beam FIB/SEM (FEI Helios Nanolab 600 FIB/SEM) with a gallium liquid metal source ion source (LMIS) operated at an accelerating voltage of 5–30kV. Initially, a ~ 100 nm layer of protective carbon was deposited using the electron beam (5 kV, 1.4 nA) on a 2 μm × 10 μm area of interest using a gas injection system (GIS) through decomposition of a phenanthrene precursor gas. A ~ 1 μm protective platinum layer was then deposited on top of the carbon using the ion beam (30 kV, 93 pA) through decomposition of a (methylcyclopentadienyl)-trimethyl platinum precursor gas. Next, two trenches were cut (30 kV, 6.5 nA) and edged-cleaned at slightly lower currents (30 kV, 2.8 nA) to allow for a roughly 1.5 μm thick lamella. Following an *in situ* lift-out procedure, a tungsten micromanipulator (Oxford Instruments) was then welded onto the lamella using platinum, and the sample was cut loose from the bulk material. After mounting the lamella as a flag onto one of the four posts of a TEM Cu half-grid (Ted Pella), the lamella was thinned in a 5 μm wide window (5 kV, 81 pA) and cleaned at low voltage and current (2 kV, 28 pA) until a final thickness of roughly 20–80 nm was achieved near the surface of the lamella.

### Scanning transmission electron microscopy (STEM) with energy dispersive spectroscopy (STEM-EDS) and electron energy loss spectroscopy (STEM-EELS)

Imaging of enamel specimens was performed using an JEOL GrandARM 300F with a cold-cathode field-emission electron gun used at an accelerating voltage of 300 kV, using a probe current of ~ 204 pA with a dwell time of 10 μs. The collection semi-angle used was 106–180 mrad for high-angle annular dark-field (HAADF) imaging. Elemental maps were recorded using EDS using a windowless 100 mm^2^ Xmax^N^ 100TLE Silicon Drift detector (SDD) with a solid angle of approximately 0.98 sr (Oxford Instruments NanoAnalysis) with a resolution of 1024 × 1024 pixels with a dwell time of 10 μs per pixel. Elemental maps were binned (4×4) and converted to mole fractions, using QuantMap (AZtecTEM). Binned mole fraction maps were then exported for further processing and visualization using Matlab 2022b (Mathworks, Natick, MA).

Line profiles (mole fractions as a function of distance in the direction normal to the external enamel surface) were determined by resampling regions of interest (ROIs) within elemental maps (determined by EDS) on a rectangular query grid rotated such that the y-direction was normal to the interface, as assessed from Ca maps. Resampling by linear interpolation was carried out using the griddedInterpolant() function included in Matlab r2022b (Mathworks, Natick, MA). Resampled ROIs were then averaged in the direction parallel to the interface. The position of the outer surface in treated and untreated samples, of the interface between the Fe and Sn rich layer and underlying enamel were identified manually from line profiles. Profiles were aligned on the outer surface position, and the distance axis was set to zero at the interface between the Fe and Sn rich layer and enamel. Data were plotted as the mean value at the given distance (solid circles), and in smoothed form (lines), as the local 3-point mean (moving average with span 3, using the movmean() function).

EEL spectra were acquired with a GIF continuum system (Gatan) using a K3 IS direct electron detector (Gatan) in counting mode at 300 kV. The high quantum efficiency of this detector (DQE up to 90%) allowed the simultaneous acquisition of the relevant inner shell ionization (core loss) edges and zero loss region at high energy resolution, except for the phosphorous K and L edges, which were outside the selected energy range. The convergence semi-angle of the probe was 19 mrad, and the probe current was ~ 27 pA, as determined using a Faraday cup. The collection semi-angle of 36 mrad was defined by the EELS entrance aperture (5 mm). The three-dimensional spectrum image dataset was collected using an energy dispersion of 0.35 eV/channel and the probe dwell time was 4 ms/pixel with a pixel size of 6 nm, with sub-pixel scanning enabled (32 × 32) to yield a ~ 3.8 Å pixel. Simultaneously, ADF images were acquired using a collection semi-angle of 51–115mrad. In post-processing, the zero-loss peak was aligned in every pixel of the spectrum image using GMS software (Gatan, Inc). Elemental Quantification Analysis was performed in the same software, using a Hartree-Slater cross-section model and including plural scattering corrections.

### High-resolution TEM (HRTEM) imaging

HRTEM imaging of enamel specimens was performed using an JEOL GrandARM 300F at an accelerating voltage of 300 kV. Images (edge length: 4096 pixels, scale factor 0.0328 nm/pixel) were processed using Matlab r2022b (Mathworks, Natick, MA). Two-dimensional Fourier transforms of regions of interest (edge length: 1024 pixels) were determined using fft2() and rearranged using fftshift() to move the zero frequency components to the center of the image. Fourier transform images were unwrapped in the azimuthal direction by interpolation using griddedInterpolant() with a query grid in polar coordinates (radial pitch: 0.0298 nm^−1^/pixel; azimuthal pitch: 1°/pixel) and integrated in the azimuthal direction.

### X-ray photoelectron spectroscopy (XPS)

Two mandibular (M1) rat molars, one from Fer + SnF_2_ treated group and one from control group, were dissected and attached using copper tape (Electron Microscopy Sciences). XPS analysis was conducted using a Thermo Scientific Nexsa G2 using an Al-Ka X-ray source, with the following parameters: pressure of 2·10^−9^ torr (2.5·10^−7^ Pa), an X-ray gun power of 150 W, a spot diameter of 100 μm, and a takeoff angle of 0°. XPS survey spectra were acquired under a pass energy of 100 eV, using a step size of 1 eV. High-resolution spectra for F, Fe, Ca, P, O, Sn, Na, and Mg were acquired under a pass energy of 50 eV, using a step size of 0.1 eV, and averaging over 10 scans. For depth profiling, the surface was excavated using an argon ion beam (4 keV, diameter 500 μm, ‘high current’ mode, 30–300s increment) between successive spectra. All data were processed using Avantage (Thermo Scientific), and spectra were referenced to adventitious carbon at 284.8 eV.

### 16S rRNA sequencing

Cells were pelleted from dental plaque by centrifuging at maximum speed for 5 min. DNA was extracted from the pellets using the Qiagen DNeasy PowerSoil htp kit according to the manufacturer’s instructions within a sterile class II laminar flow hood. Mock washes and mock extractions were included to control for microbial DNA contamination arising through the sonication and extraction processes, respectively.

Polymerase chain reaction (PCR) amplification of V1-V2 region of 16S rRNA gene was performed using Golay-barcoded universal primers 27F and 338R. Four replicate PCR reactions were performed for each sample using Q5 Hot Start High Fidelity DNA Polymerase (New England BioLabs). Each PCR reaction contained: 4.3 μl microbial DNA-free water, 5 μl 5X buffer, 0.5 μl dNTPs (10 mM), 0.17 μl Q5 Hot Start Polymerase, 6.25 μl each primer (2μM), and 2.5 μl DNA. PCR reactions with no added template or synthetic DNAs were performed as negative and positive controls, respectively^55^. PCR amplification was done on a Mastercycler Nexus Gradient (Eppendorf) using the following conditions: DNA denaturation at 98 °C for 1 min, then 20 cycles of denaturation at 98 °C for 10 sec, annealing 56 °C for 20 sec and extension 72 °C for 20 sec, last extension was at 72 °C for 8 min. PCR replicates were pooled and then purified using a 1:1 ratio of Agencourt AMPure XP beads (Beckman Coulter, Indianapolis, IN), following the manufacturer’s protocol. The final library was prepared by pooling 10 μg of amplified DNA per sample. Those that did not arrive at the DNA concentration threshold (e.g., negative control samples) were incorporated into the final pool by adding 12 μl. The library was sequenced to obtain 2×250 bp paired-end reads using the MiSeq Illumina^56^.

To analyze 16S RNA gene sequences, we used QIIME2 v19.4^57^. We obtained taxonomic assignments based on GreenGenes 16S rRNA database v.13_8^58^ and ASV analysis of shared and unique bacterial taxa through DADA2^59^. PCoA was performed using library ape for R programming language^60^. To test the differences between communities, we used library vegan and UniFrac distances (https://CRAN.R-project.org/package=vegan). R environment (version 4.0.3) was used for statistical analysis. Non-parametrical test Wilcoxon Rank Sum Test was performed for the pairwise comparison between treatment groups for richness and Shannon diversity analysis. PERMANOVA analysis was performed for weighted UniFrac principal coordinate analysis to evaluate the differences between treatment groups. Statistical significance was considered < 0.05.

### Statistical analysis

The data presented as the mean ± standard deviation were performed at least three times independently unless otherwise stated. One-way analysis of variance (ANOVA) followed by the Tukey test was used to determine the statistical significance between the control and the experimental groups unless otherwise stated. p values < 0.05 were considered statistically significant.

## Data Availability

16S rRNA sequencing data is available in the public repository NCBI under the accession number PRJNA914620. All the other data that support the findings of this study are available in the main text or the supplementary materials.
